# The associations between cigarette smoking and health-related behaviors among Chinese school-aged adolescents

**DOI:** 10.1186/s12971-017-0132-0

**Published:** 2017-06-02

**Authors:** Meng Wang, Hao Wang, Fang-Rong Fei, Chun-Xiao Xu, Xiao-Fu Du, Jie-Ming Zhong

**Affiliations:** Zhejiang Provincial Center for Disease Control and Prevention, 3399 Binsheng Road, Hangzhou, 310051 China

**Keywords:** Cigarette smoking, Adolescents, Diet, Physical activity, Behavior

## Abstract

**Background:**

Evidence on the interrelations between cigarette smoking and a cluster of lifestyle behaviors is scarce for the Chinese youth population. This study is conducted to identify the associations between cigarette smoking and multiple health-related behaviors in a Chinese sample of adolescents.

**Methods:**

We used data from 2012 Zhejiang Youth Risk Behavior Survey, which is a school-based survey of 19,542 adolescents that assess risk behaviors using a self-reported questionnaire. The interrelations of cigarette smoking with lifestyle behaviors were investigated using logistic regression models.

**Results:**

Cigarette smoking was significantly inversely associated with breakfast (AOR = 0.58), vegetables (AOR = 0.81), fruits (AOR = 0.81), milk consumption (AOR = 0.69) and attending physical education classes (AOR = 0.69), while positively associated with soft drinks (AOR = 2.05), fast food consumption (AOR = 1.21), muscle strengthening activity (AOR = 1.67), computer use (AOR = 1.93) and alcohol drinking (AOR = 5.40).

**Conclusions:**

The results suggested that cigarette smoking was associated with a cluster of health-related behaviors in adolescents, which should be considered in health promotion interventions to target multiple health behaviors.

**Electronic supplementary material:**

The online version of this article (doi:10.1186/s12971-017-0132-0) contains supplementary material, which is available to authorized users.

## Background

The tobacco epidemic is one of the biggest public health threats worldwide. In 2013, tobacco smoke was the second leading contributor to the global disease burden, accounting for 6.1 million deaths and 143.5 million disability-adjusted life-years (DALYs) [1]. Nearly 80% of the more than 1 billion smokers worldwide live in low-and middle-income countries [2]. With 1.3 billion citizens and 301 million adult current smokers [3], China is the largest tobacco consuming country. In addition to the high smoking prevalence among adults, it is worth noting that cigarette smoking has gained popularity in Chinese adolescents. According to the 2014 Global Youth Tobacco Survey (GYTS), the percentages of ever cigarette smokers and current cigarette smokers among adolescents aged 13–15 years in China were 17.9% and 5.9% [4].

Smoking is not only a modifiable health-related behavior that can have negative impacts on the physical development of adolescents. Adolescent smokers are at increased risk for atherosclerosis, asthma and have worse bone mineral density [5–7]. Smoking is also reported to be linked to lifestyle behaviors, involving diet, as well as alcohol consumption, physical activity, and sleep [8–10]. However, most studies tend to investigate the interrelations between smoking and one or several lifestyle behaviors, and evidence on the associations of smoking with a cluster of health-related behaviors is scarce. Adolescence is a critical period in the life course when lifestyle patterns establish and track into adulthood. To develop health promotion interventions to address behaviors in adolescents, it is imperative to identify the associations between cigarette smoking and a cluster of health-related behaviors [8]. In the present study, we aim to explore the interrelations between cigarette smoking and multiple health-related behaviors (dietary, physical activity, sedentary activity, alcohol drinking and sleep) in Chinese adolescents.

## Methods

### Data source

The data presented here were obtained from 2012 Zhejiang Youth Risk Behavior Survey (YRBS), which is a school-based survey of adolescents that assess risk behaviors. In total, 20,589 middle and high school students agreed to participate in the survey, yielding a response rate of 97.53%. After excluding 108 ineligible questionnaires and 939 students who were not the natives of Zhejiang Province, finally, 19,542 students were recruited in the present study. A multistage, stratified cluster sampling technique was used. In the first stage, 30 counties were sampled from all 90 counties of Zhejiang Province on the basis of socioeconomic status. Then, schools in selected counties were stratified according to their levels (middle school, academic and vocational high school) and geographical positions (from west to east, from north to south). Then, based on the number of students in each level of school, samples of classes were selected and students were invited to complete a self-administered questionnaire. The self-administered questionnaire derived from U.S. 1991–2015 Youth Risk Behavior Surveillance System (YRBSS) and Global School-based Student Health Survey (GSHS) and was used to access the cigarette smoking, diet, as well as physical activity, sedentary activity, alcohol drinking, and sleep behaviors. Without teachers present, students completed the anonymous questionnaire in the classroom independently. After finished, questionnaires were collected by the researchers. To make all the participants voluntary, parents / guardians of the selected students and the school officials were sent a written letter to inform them that a study was to be conducted to examine issues relevant to adolescent health, and given the option to refuse the students’ participation in the study. Consent was obtained from parents / guardians of the selected students to publish the collected data. Besides, all the researchers were strictly trained to protect the students’ privacy and ensure the confidentiality of the personal data. In particular, our study abided by the “Declaration of Helsinki” and was approved by the ethnics committee of Zhejiang Provincial Center for Disease Control and Prevention. More detailed information about the study was provided elsewhere [11].

### Variables definition

The participants’ cigarette smoking behavior was accessed by the question: “During the past 30 days, on how many days did you smoke cigarettes? (0 days, 1–2 days, 3–5 days, 6–9 days, 10–19 days, 20–29 days, 30 days)”. Participants were considered as current smokers if they answered had smoked at least 1 day during the past 30 days. Other questions involved diet, as well as physical activity, sedentary activity, alcohol drinking, and sleep and are described in Table 1. Some previously identified smoking variables were included: age range (≤ 13, 14, 15, ≥ 16 years), sex (girls, boys), location of school (urban, rural), and school level (middle school, academic high school, vocational high school). Besides, possible interaction between the clustered factors (fruits and vegetables; fast food and soft drinks; watch TV and use computer) and other health-related behaviors was tested and these significant were also taken into consideration in this study (Additional file 1: Table S1)

**Table 1:** The definition of some variables in the 2012 Zhejiang Youth Risk Behavior Survey

Behaviors	Definition
Dietary	
Breakfast (Daily)	During the past 30 days, in an average week, how many days did you eat breakfast? with five answer options, “0 day”, “1–2 days”, “3–4 days”, “5–6 days”, “7 days”. The answers were further classified into three groups, “Never” (0 day), “sometimes” (1–2 days, 3–4 days, and 5–6 days), and “daily” (7 days).
Fruits (≥2 times/d)	During the past 30 days, how many times did you eat fruits every day? (0 time, <1 time, 1 time, 2 times, 3 times, 4 times, ≥ 5 times)
Vegetables (≥2 times/d)	During the past 30 days, how many times did you eat vegetables every day? (0 time, <1 time, 1 time, 2 times, 3 times, 4 times, ≥ 5 times)
Milk (≥3 d/wk)	During the past 30 days, how many days did you drink milk every week? (0 day, <1 day, 1–2 days, 3–4 days, 5–7 days)
Soft drinks (≥1 times/d)	During the past 7 days, how many times did you drink soft drinks, such as Coke, Pepsi, or Sprite? (0 time, 1–3 times, 4–6 times, once a day, twice a day, three times a day, ≥ 4 times a day)
Fast food (≥2 d/wk)	During the past 7 days, how many days did you eat fast food, such as hamburger, hotdogs, or potato chips, etc.? (0 day, 1 day, 2 days, 3 days, 4 days, 5 days, 6 days, 7 days)
Physical activity	
Moderate physical activity(≥2d/wk)	During the past 7 days, how many days did you participate in at least 60 min of any kind of physical activity that increased your heart rate and made you breathe hard, such as kicking shuttlecock, fast bicycling, and doing housework, etc.? (0 day, 1 day, 2 days, 3 days, 4 days, 5 days, 6 days, 7 days)
Muscle strengthening activity (≥2 d/wk)	During the past 7 days, how many days did you do exercises to strengthen or tone your muscles, such as push-ups, sit-ups, or weight lifting, etc.? (0 day, 1 day, 2 days, 3 days, 4 days, 5 days, 6 days, 7 days)
Attend physical education classes (≥2 d/wk)	In an average week when you are in school, on how many days do you go to physical education classes? (0 day, 1 day, 2 days, 3 days, 4 days, 5 days, 6 days, 7 days)
Sedentary Activity	
Watch TV (≥2 h/d)	On an average school day, how many hours do you watch TV? (0 h, <1 h, 1–2 h, 2–3 h, 3–4 h, 4–5 h, ≥5 h)
Use computer (≥2 h/d)	During the past 7 days, in an average day, on how many hours did you spend using a computer for playing games, surfing, e-mailing, or watching movies, etc.? (open question)
Other	
Sleep duration (≥8 h/d)	During the past 30 days, how many hours did you spend on sleeping every day? (open question)
Drink alcohol (yes)	During the past 30 days, on how many days did you have at least one drink of alcohol? (0 days, 1–2 days, 3–5 days, 6–9 days, 10–19 days, 20–29 days, 30 days)”. Participants who answered had drunk alcohol at least 1 day during the past 30 days were considered to have the alcohol drinking behavior

### Statistical analysis

The associations between current smoking and demographic characteristics of sex, location of school, and school level were tested with Chi-square tests. For age, the association with current smoking was tested with linear-by-linear association Chi-square test. In the analysis on the association of current smoking and other health-related behaviors, logistic regression analyses were conducted by three steps in sequence. First, univariate logistic regression analyses were conducted to preliminarily assess the association. Second, a multivariate logistic regression model was constructed by adding all the variables. Third, two multivariate logistic regression analyses were conducted to determine the effects of current smoking on health-related behaviors in different sex and location of school. Besides, another logistic regression analysis process was repeated to further analyze each grade effect of cigarette number on other health-related behaviors. In this section, the number of cigarettes smoked was categorized as ≤1 cigarette per day, 2–10 cigarettes per day and >10 cigarettes per day. The effect values were reported by odds ratio (OR) and adjusted odds ratio (AOR) with their 95% confidence intervals (CIs) and a value of <0.05 was considered to be statistically significant. All analyses were performed using SAS statistical package (version 9.2, SAS Institute, Inc., Cary, NC, USA).

## Results

Table 2 showed that the demographic characteristics of sex, age, location of school and school level were associated with current smoking. The prevalence of smoking was statistically significantly higher among boys, as well as older students, rural school students and vocational high school students.

**Table 2:** Demographic characteristics of students from 2012 Zhejiang Youth Risk Behavior Survey and the associations with smoking status

Characteristics	*N* (%)	Smoking status		Chi-square	*P*
		Nonsmokers (*N* = 17,993)	Current smokers (*N* = 1549)		
Sex				805.2	<0.001
Boys	9723 (49.75)	8416 (86.56)	1307 (13.44)		
Girls	9819 (50.25)	9577 (97.54)	242 (2.46)		
Age range (years)				304.7*	<0.001*
≤ 13	3904 (19.98)	3797 (97.26)	107 (2.74)		
14-	3038 (15.54)	2894 (95.26)	144 (4.74)		
15-	3302 (16.90)	3026 (91.64)	276 (8.36)		
≥ 16	9298 (47.58)	8276 (89.01)	1022 (10.99)		
Location of school				26.3	<0.001
Urban	7346 (37.59)	6858 (93.36)	488 (6.64)		
Rural	12,196 (62.41)	11,135 (91.30)	1061 (8.70)		
School level				659.3	<0.001
Middle school	9617 (49.21)	9175 (95.40)	442 (4.60)		
Academic high school	5495 (28.12)	5140 (93.54)	355 (6.46)		
Vocational high school	4430 (22.67)	3678 (83.02)	752 (16.98)		

*Linear-by-linear association chi-square test results

Tables 3 and 4 showed the associations between current smoking and other health-related behaviors. Compared to nonsmokers, current smokers had significant (19%-42%) reduction in having a daily breakfast, eating fruits and vegetables at least twice in a day, and milk at least three days in a week. Stratified by sex and location of school, girls were less likely to have a daily breakfast, eat fruits and vegetables at least twice in a day; urban school students were less likely to eat fruits and vegetables at least twice in a day, and milk at least three days in a week. Current smokers were 2.05 times (AOR = 2.05) more likely to consume soft drinks at least once in a day and 1.21 times (AOR = 1.21) more likely to consume fast food at least two days in a week as compared to nonsmokers. Girls were more likely to consume soft drinks at least once in a day and fast food at least two days in a week. Current smoking was significantly associated with increased probability of muscle strengthening activity, and decreased probability of attending physical education classes at least two days in a week. Urban school students were more likely to attend muscle strengthening activity and less likely to attend physical education classes at least two days in a week. Current smokers were 1.93 times (AOR = 1.93) more likely to use computer at least two hours in a day as compared to nonsmokers and boys and urban school students were more likely to use computer at least two hours in a day. Current smoking was significantly associated with increased probability of alcohol drinking (AOR = 5.38) and girls and rural school students were more likely to drink alcohol.

**Table 3:** Smoking status and its associations with health-related behaviors among all students from 2012 Zhejiang Youth Risk Behavior Survey

Behaviors	Smoking status^a^	All students	
		OR (95% CI)	AOR^c^ (95% CI)
Dietary			
Breakfast (Daily)^a^	Nonsmokers	Ref.	Ref.
	Current smokers	**0.39 (0.35–0.43)**	**0.58 (0.52–0.66)**
Fruits (≥2 times/d)^a^	Nonsmokers	Ref.	Ref.
	Current smokers	**0.79 (0.71–0.89)**	**0.81 (0.71–0.91)**
Vegetables (≥2 times/d)^a^	Nonsmokers	Ref.	Ref.
	Current smokers	**0.67 (0.60–0.75)**	**0.81 (0.71–0.91)**
Milk (≥3 d/wk)^a^	Nonsmokers	Ref.	Ref.
	Current smokers	**0.60 (0.54–0.67)**	**0.69 (0.62–0.77)**
Soft drinks (≥1 times/d)^b^	Nonsmokers	Ref.	Ref.
	Current smokers	**2.92 (2.57–3.33)**	**2.05 (1.77–2.36)**
Fast food (≥2 d/wk)^b^	Nonsmokers	Ref.	Ref.
	Current smokers	**2.02 (1.77–2.30)**	**1.21 (1.05–1.40)**
Physical Activity			
Moderate physical activity (≥2d/wk)^b^	Nonsmokers	Ref.	Ref.
	Current smokers	0.97 (0.87–1.08)	0.92 (0.81–1.04)
Muscle strengthening activity (≥2 d/wk)^b^	Nonsmokers	Ref.	Ref.
	Current smokers	**1.63 (1.47–1.81)**	**1.67 (1.49–1.88)**
Attend physical education classes (≥2 d/wk)	Nonsmokers	Ref.	Ref.
	Current smokers	**0.56 (0.49–0.64)**	**0.69 (0.60–0.80)**
Sedentary Activity			
Watch TV (≥2 h/d)	Nonsmokers	Ref.	Ref.
	Current smokers	**1.22 (1.09–1.37)**	0.91 (0.80–1.03)
Use computer (≥2 h/d)^b^	Nonsmokers	Ref.	Ref.
	Current smokers	**2.96 (2.64–3.31)**	**1.93 (1.71–2.18)**
Other			
Sleep duration (≥8 h/d)^a^	Nonsmokers	Ref.	Ref.
	Current smokers	0.99 (0.88–1.11)	1.04 (0.97–1.11)
Drink alcohol (yes)^a^	Nonsmokers	Ref.	Ref.
	Current smokers	**7.27 (6.49–8.13)**	**5.38 (4.79–6.05)**

Bold numbers represent significant results

^a^During the past 30 days

^b^During the past 7 days

^c^Adjusted for all the covariates listed in the table

**Table 4:** Smoking status and its associations with health-related behaviors stratified by sex and location of school among students from 2012 Zhejiang Youth Risk Behavior Survey

Behaviors	Smoking status^a^	Sex		Location of school	
		Boys	Girls	Urban	Rural
		AOR^c^ (95% CI)	AOR^c^ (95% CI)	AOR^c^ (95% CI)	AOR^c^ (95% CI)
Dietary					
Breakfast (Daily)\^a^	Nonsmokers	Ref.	Ref.	Ref.	Ref.
	Current smokers	**0.58 (0.51–0.65)**	**0.50 (0.37–0.66)**	**0.65 (0.53–0.79)**	**0.57 (0.49–0.65)**
Fruits (≥2 times/d)^a^	Nonsmokers	Ref.	Ref.	Ref.	Ref.
	Current smokers	0.91 (0.79–1.05)	0.81 (0.60–1.09)	**0.58 (0.46–0.72)**	0.94 (0.81–1.10)
Vegetables (≥2 times/d)^a^	Nonsmokers	Ref.	Ref.	Ref.	Ref.
	Current smokers	**0.83 (0.73–0.95)**	**0.65 (0.50–0.86)**	**0.77 (0.63–0.94)**	**0.84 (0.73–0.96)**
Milk (≥3 d/wk)^a^	Nonsmokers	Ref.	Ref.	Ref.	Ref.
	Current smokers	**0.66 (0.58–0.75)**	0.81 (0.62–1.07)	**0.64 (0.52–0.78)**	**0.73 (0.64–0.84)**
Soft drinks (≥1 times/d)^b^	Nonsmokers	Ref.	Ref.	Ref.	Ref.
	Current smokers	**1.67 (1.42–1.95)**	**1.87 (1.28–2.73)**	**2.26 (1.78–2.87)**	**2.00 (1.67–2.40)**
Fast food (≥2 d/wk)^b^	Nonsmokers	Ref.	Ref.	Ref.	Ref.
	Current smokers	**1.37 (1.15–1.63)**	**1.61 (1.18–2.19)**	1.18 (0.93–1.51)	**1.28 (1.06–1.54)**
Physical Activity					
Moderate physical activity (≥2d/wk)^b^	Nonsmokers	Ref.	Ref.	Ref.	Ref.
	Current smokers	0.91 (0.79–1.05)	**0.68 (0.52–0.90)**	0.86 (0.69–1.06)	0.96 (0.83–1.11)
Muscle strengthening activity (≥2 d/wk)^b^	Nonsmokers	Ref.	Ref.	Ref.	Ref.
	Current smokers	1.10 (0.97–1.26)	**1.55 (1.12–2.12)**	**1.79 (1.45–2.22)**	**1.65 (1.43–1.91)**
Attend physical education classes (≥2 d/wk)	Nonsmokers	Ref.	Ref.	Ref.	Ref.
	Current smokers	**0.65 (0.55–0.77)**	0.75 (0.53–1.04)	**0.53 (0.42–0.66)**	**0.79 (0.64–0.96)**
Sedentary Activity					
Watch TV (≥2 h/d)	Nonsmokers	Ref.	Ref.	Ref.	Ref.
	Current smokers	0.97 (0.84–1.12)	1.20 (0.89–1.60)	0.84 (0.66–1.07)	0.90 (0.77–1.05)
Use computer (≥2 h/d)^b^	Nonsmokers	Ref.	Ref.	Ref.	Ref.
	Current smokers	**2.07 (1.81–2.37)**	**1.41 (1.05–1.88)**	**2.35 (1.88–2.93)**	**1.73 (1.50–2.00)**
Other					
Sleep duration (≥8 h/d)^a^	Nonsmokers	Ref.	Ref.	Ref.	Ref.
	Current smokers	0.99 (0.86–1.14)	0.96 (0.73–1.28)	1.18 (0.96–1.45)	0.93 (0.80–1.08)
Drink alcohol (yes)^a^	Nonsmokers	Ref.	Ref.	Ref.	Ref.
	Current smokers	**4.49 (3.94–5.11)**	**10.62 (7.67–14.72)**	**4.67 (3.79–5.75)**	**5.80 (5.03–6.69)**

Bold numbers represent significant results

^a^During the past 30 days.

^b^During the past 7 days

^c^Adjusted for all the covariates listed in the table

Additional file 2: Table S2 showed the number of cigarettes smoked per day was significantly associated with some health-related behaviors and dose-response relationships were observed. From ≤1 cigarette per day to >10 cigarettes per day, decreasing AORs were observed in the breakfast, milk consumption, and attending physical education classes and increasing AORs were observed in soft drinks consumption, as well as muscle strengthening activity and alcohol drinking. Dose-response relationships existed by sex and location (Additional file 3: Table S3).

## Discussion

According to the present study, significantly higher prevalence of current smoking was observed in boys, as well as in older, rural school and vocational high school students. These results were in agreement with previous data [12–14]. We were able to confirm the clustering of cigarette smoking with multiple lifestyle behaviors in adolescents and the cluster patterns were different according to sex and location of school. Dose-response effects of smoking status (from ≤1 cigarette per day to 2–10 cigarettes per day to >10 cigarettes per day) on lifestyle behaviors were also observed in the current study.

A less healthy diet (reduced consumption of breakfast, vegetables, fruits, milk and increased consumption of soft drinks, fast food) was found among current smokers as compared to nonsmokers. These results reinforced previous findings that adolescent smoking was inversely associated with healthy dietary behaviors [8, 15–17]. In recent years, there have been considerable studies on the negative impact of cigarette smoking on physical activity among children and adolescents [18, 19]. We showed that adolescent smokers were significantly less likely to attend physical education classes than were nonsmokers, which supported the previous researchers [18, 19]. However, a positive association between cigarette smoking and muscle strengthening activity was observed. Further studies were warranted to confirm and explore the nature of this association. There have been few studies exploring the association between cigarette smoking and sedentary behaviors. Giannakopoulos et al. [20] reported that adolescent smokers spend more time watching TV and playing videogames than adolescent non-smokers. Our results confirmed that smoking adolescents spend longer time on computer. There is evidence that smoking and alcohol drinking behaviors tend to cluster together in adolescents [21, 22]. In our study, it was found that cigarette smoking was strongly associated with increased risk of drinking alcohol, which has been reported in previous studies [10, 23–26]. Overall, our findings also indicated that the effects of smoking on a series of health-related behaviors of adolescents were modified by sex and school location. One possible reason, we hypothesized, was the different characteristics of health-related behaviors in these groups. Take breakfast consumption behavior for example, one of our studies, with the same participants as the current study, showed that the prevalence of daily breakfast consumption was significantly lower among rural schools than urban schools [11], which may partly explain our findings of reduced breakfast consumption among rural current smokers as compared to urban current smokers. Dose-response relationships between smoking frequency and some certain behaviors (e.g. watching television and video, intake of fast food) have been reported in previous studies [8, 27]. The results of our study confirmed the dose-response effects of smoking status on lifestyle behaviors. However, due to the measurement differences, our data on the dose-response effects of smoking among adolescents were not comparable to those in previous studies.

The results from our study were subject to several limitations. First, as this study was carried out with a cross-sectional design, we could not provide causal associations of cigarette smoking with other health-related behaviors. Second, the generalization of our results to Chinese adolescent population is under discussion as the sample was selected in one province of China. Third, the smoking behavior in adolescents was self-reported through a single item rather than biomarker of smoking.

## Conclusions

In conclusion, we reported the interrelations between cigarette smoking and multiple lifestyle behaviors in adolescents. These associations should be considered in the planning of health promotion interventions to target multiple health behaviors.

## Additional files

Additional file 1: Table S1. Possible interaction between clustered factors (fruits and vegetables; fast food and soft drinks; watch TV and use computer) and other behaviors. (DOCX 20 kb)

Additional file 2: Table S2. Number of cigarettes smoked per day and its associations with health-related behaviors among all students. (DOCX 18 kb)

Additional file 3: Table S3. Number of cigarettes smoked per day and its associations with health-related behaviors stratified by sex and location of school. (DOCX 23 kb)

## Abbreviations

AOR: adjusted odds ratio
CI: confidence interval
GYTS: Global youth tobacco survey
OR: odds ratio
