# Supplementary material for: The associations between cigarette smoking and health-related behaviors among Chinese school-aged adolescents
Source: Tob Induc Dis. 2017 Jun 2;15:27. doi: 10.1186/s12971-017-0132-0 (PMC5457567; doi:10.1186/s12971-017-0132-0)
Supplement: Supplementary file 1 — Possible interaction between clustered factors (fruits and vegetables; fast food and soft drinks; watch TV and use computer) and other behaviors. (DOCX 20 kb) [file 12971_2017_132_MOESM1_ESM.docx]

| **Additional file 1: Table S1.** Possible interaction between clustered factors (fruits and vegetables; fast food and soft drinks; watch TV and use computer) and other behaviors | | | | | | | | | | | | | | | | | | | |
| --- | --- | --- | --- | --- | --- | --- | --- | --- | --- | --- | --- | --- | --- | --- | --- | --- | --- | --- | --- |
| **Dietary** | All Students (P value) | | |  | Boys (P value) | | |  | Girls (P value) | | |  | Urban school students (P value) | | |  | Urban school students (P value) | | |
|  | Fruits*  vegetables | Soft drinks*  fast food | Watch TV*  use computer |  | Fruits*  vegetables | Soft drinks*  fast food | Watch TV*  use computer |  | Fruits*  vegetables | Soft drinks*  fast food | Watch TV*  use computer |  | Fruits*  vegetables | Soft drinks*  fast food | Watch TV*  use computer |  | Fruits*  vegetables | Soft drinks*  fast food | Watch TV*  use computer |
| Breakfast (Daily) **†** | <0.001 | <0.001 | <0.001 |  | <0.001 | <0.001 | <0.001 |  | <0.001 | <0.001 | <0.001 |  | <0.001 | <0.001 | <0.001 |  | <0.001 | <0.001 | <0.001 |
| Fruits (≥2 times/d)**†** | NA | <0.001 | <0.001 |  | NA | <0.001 | <0.001 |  | NA | <0.001 | <0.001 |  | NA | <0.001 | <0.001 |  | NA | <0.001 | <0.001 |
| Vegetables (≥2 times/d)**†** | NA | 0.07 | 0.16 |  | NA | 0.16 | 0.22 |  | NA | 0.33 | 0.46 |  | NA | 0.002 | 0.001 |  | NA | 0.99 | 0.31 |
| Milk (≥3 d/wk)**†** | <0.001 | <0.001 | <0.001 |  | <0.001 | <0.001 | <0.001 |  | <0.001 | 0.05 | <0.001 |  | <0.001 | 0.01 | <0.001 |  | <0.001 | <0.001 | <0.001 |
| Soft drinks (≥1 times/d)**‡** | <0.001 | NA | <0.001 |  | <0.001 | NA | <0.001 |  | <0.001 | NA | <0.001 |  | 0.04 | NA | <0.001 |  | <0.001 | NA | <0.001 |
| Fast food (≥2 d/wk)**‡** | <0.001 | NA | <0.001 |  | <0.001 | NA | <0.001 |  | <0.001 | NA | <0.001 |  | 0.02 | NA | <0.001 |  | <0.001 | NA | <0.001 |
| **Physical Activity** |  |  |  |  |  |  |  |  |  |  |  |  |  |  |  |  |  |  |  |
| Moderate physical activity (≥2d/wk)‡ | <0.001 | <0.001 | 0.58 |  | <0.001 | 0.002 | 0.19 |  | <0.001 | 0.02 | 0.16 |  | <0.001 | 0.001 | 0.01 |  | <0.001 | <0.001 | 0.01 |
| Muscle strengthening activity (≥2 d/wk)**‡** | <0.001 | <0.001 | 0.06 |  | <0.001 | <0.001 | 0.01 |  | <0.001 | <0.001 | 0.10 |  | <0.001 | <0.001 | 0.03 |  | <0.001 | <0.001 | 0.74 |
| Attend physical education classes (≥2 d/wk) | <0.001 | 0.06 | <0.001 |  | <0.001 | 0.03 | 0.06 |  | <0.001 | 0.48 | <0.001 |  | <0.001 | 0.08 | <0.001 |  | <0.001 | 0.89 | 0.50 |
| **Sedentary Activity** |  |  |  |  |  |  |  |  |  |  |  |  |  |  |  |  |  |  |  |
| Watch TV  (≥2 hours/d) | <0.001 | <0.001 | NA |  | <0.001 | <0.001 | NA |  | <0.001 | <0.001 | NA |  | <0.001 | <0.001 | NA |  | <0.001 | <0.001 | NA |
| Use computer (≥2 hours/d)**‡** | 0.26 | <0.001 | NA |  | 0.31 | <0.001 | NA |  | 0.33 | <0.001 | NA |  | 0.002 | <0.001 | NA |  | <0.001 | <0.001 | NA |
| **Other** |  |  |  |  |  |  |  |  |  |  |  |  |  |  |  |  |  |  |  |
| Sleep duration (≥8 hours/d)**†** | <0.001 | 0.35 | <0.001 |  | <0.001 | 0.23 | <0.001 |  | <0.001 | 0.09 | <0.001 |  | <0.001 | 0.12 | <0.001 |  | <0.001 | 0.001 | <0.001 |
| Drink alcohol (yes)**†** | <0.001 | <0.001 | <0.001 |  | 0.002 | <0.001 | <0.001 |  | 0.12 | <0.001 | <0.001 |  | 0.002 | <0.001 | <0.001 |  | 0.01 | <0.001 | <0.001 |
| NA: not available  **†** During the past 30 days. **‡** During the past 7 days. | | | | | | | | | | | | | | | | | | | |
