# Supplementary material for: The associations between cigarette smoking and health-related behaviors among Chinese school-aged adolescents
Source: Tob Induc Dis. 2017 Jun 2;15:27. doi: 10.1186/s12971-017-0132-0 (PMC5457567; doi:10.1186/s12971-017-0132-0)
Supplement: Supplementary file 2 — Number of cigarettes smoked per day and its associations with health-related behaviors among all students. (DOCX 18 kb) [file 12971_2017_132_MOESM2_ESM.docx]

| **Supplementary table 2.** Number of cigarettes smoked per day and its associations with health-related behaviors among all students | | | | |
| --- | --- | --- | --- | --- |
| Behaviors | Number of cigarettes smoked**†** | All students | | |
|  |  |  |  |  |
|  |  | OR (95% CI) | AOR (95% CI) | |
| **Dietary** |  |  |  | |
| Breakfast (Daily) **†** | ≤1 cigarette/d | Ref. | Ref. | |
|  | 2-10 cigarettes/d | **0.34 (0.29-0.40)** | **0.53 (0.45-0.63)** | |
|  | >10 cigarettes/d | **0.21 (0.12-0.36)** | **0.42 (0.24-0.74)** | |
| Fruits (≥2 times/d)**†** | ≤1 cigarette/d | Ref. | Ref. | |
|  | 2-10 cigarettes/d | 0.90 (0.77-1.07) | 0.92 (0.77-1.10) | |
|  | >10 cigarettes/d | 0.95 (0.57-1.58) | 0.96 (0.56-1.67) | |
| Vegetables (≥2 times/d)**†** | ≤1 cigarette/d | Ref. | Ref. | |
|  | 2-10 cigarettes/d | **0.71 (0.61-0.84)** | 0.87 (0.73-1.03) | |
|  | >10 cigarettes/d | **0.37 (0.23-0.61)** | **0.45 (0.27-0.77)** | |
| Milk (≥3 d/wk)**†** | ≤1 cigarette/d | Ref. | Ref. | |
|  | 2-10 cigarettes/d | **0.60 (0.52-0.71)** | **0.70 (0.59-0.83)** | |
|  | >10 cigarettes/d | **0.49 (0.30-0.81)** | **0.56 (0.33-0.95)** | |
| Soft drinks (≥1 times/d)**‡** | ≤1 cigarette/d | Ref. | Ref. | |
|  | 2-10 cigarettes/d | **3.51 (2.93-4.21)** | **2.39 (1.97-2.91)** | |
|  | >10 cigarettes/d | **7.55 (4.63-12.31)** | **4.15 (2.46-7.00)** | |
| Fast food (≥2 d/wk)**‡** | ≤1 cigarette/d | Ref. | Ref. | |
|  | 2-10 cigarettes/d | **2.03 (1.67-2.46)** | 1.11 (0.90-1.37) | |
|  | >10 cigarettes/d | **4.85 (2.94-8.02)** | **2.25 (1.31-3.86)** | |
| **Physical Activity** |  |  |  | |
| Moderate physical activity  (≥2d/wk)**‡** | ≤1 cigarette/d | Ref. | Ref. | |
|  | 2-10 cigarettes/d | 1.01 (0.86-1.19) | 0.98 (0.82-1.17) |  |
|  | >10 cigarettes/d | 1.26 (0.75-2.14) | 1.24 (0.69-2.22) |  |
| Muscle strengthening activity (≥2 d/wk)**‡** | ≤1 cigarette/d | Ref. | Ref. |  |
|  | 2-10 cigarettes/d | **1.61 (1.37-1.88)** | **1.53 (1.29-1.82)** |  |
|  | >10 cigarettes/d | **2.30 (1.41-3.73)** | **2.36 (1.38-4.02)** |  |
| Attend physical education classes (≥2 d/wk) | ≤1 cigarette/d | Ref. | Ref. |  |
|  | 2-10 cigarettes/d | **0.58 (0.47-0.71)** | **0.77 (0.62-0.95)** |  |
|  | >10 cigarettes/d | **0.37 (0.21-0.63)** | **0.46 (0.26-0.81)** |  |
| **Sedentary Activity** |  |  |  |  |
| Watch TV (≥2 hours/d) | ≤1 cigarette/d | Ref. | Ref. |  |
|  | 2-10 cigarettes/d | 1.09 (0.91-1.30) | **0.76 (0.63-0.92)** |  |
|  | >10 cigarettes/d | **2.07 (1.26-3.39)** | 1.32 (0.77-2.23) |  |
| Use computer (≥2 hours/d)**‡** | ≤1 cigarette/d | Ref. | Ref. |  |
|  | 2-10 cigarettes/d | **3.63 (3.03-4.34)** | **2.35 (1.95-2.85)** |  |
|  | >10 cigarettes/d | **3.33 (1.94-5.73)** | 1.44 (0.81-2.59) |  |
| **Other** |  |  |  |  |
| Sleep duration (≥8 hours/d)**†** | ≤1 cigarette/d | Ref. | Ref. |  |
|  | 2-10 cigarettes/d | 0.96 (0.81-1.31) | 0.99 (0.83-1.19) |  |
|  | >10 cigarettes/d | 0.98 (0.58-1.67) | 1.04 (0.60-1.80) |  |
| Drink alcohol (yes)**†** | ≤1 cigarette/d | Ref. | Ref. |  |
|  | 2-10 cigarettes/d | **7.86 (6.60-9.36)** | **5.41 (4.52-6.49)** |  |
|  | >10 cigarettes/d | **12.42 (6.76-22.80)** | **6.53 (3.48-12.25)** |  |
| Bold numbers represent significant results.  **†** During the past 30 days. **‡** During the past 7 days. | | | | |
