# Supplementary material for: The associations between cigarette smoking and health-related behaviors among Chinese school-aged adolescents
Source: Tob Induc Dis. 2017 Jun 2;15:27. doi: 10.1186/s12971-017-0132-0 (PMC5457567; doi:10.1186/s12971-017-0132-0)
Supplement: Supplementary file 3 — Number of cigarettes smoked per day and its associations with health-related behaviors stratified by sex and location of school. (DOCX 23 kb) [file 12971_2017_132_MOESM3_ESM.docx]

| **Additional file 3 Table S3.** Number of cigarettes smoked per day and its associations with health-related behaviors stratified by sex and location of school | | | | | | | |
| --- | --- | --- | --- | --- | --- | --- | --- |
| Behaviors | Number of cigarettes smoked**†** |  | Sex | |  | Location of school | |
|  |  |  | Boys | Girls |  | Urban | Rural |
|  |  |  | AOR (95% CI) | AOR (95% CI) |  | AOR (95% CI) | AOR (95% CI) |
| **Dietary** |  |  |  |  |  |  |  |
| Breakfast (Daily) **†** | ≤1 cigarette/d |  | Ref. | Ref. |  | Ref. | Ref. |
|  | 2-10 cigarettes/d |  | **0.50 (0.41-0.60)** | 0.63 (0.37-1.10) |  | **0.63 (0.46-0.84)** | **0.50 (0.40-0.61)** |
|  | >10 cigarettes/d |  | **0.39 (0.21-0.72)** | 0.57 (0.14-2.24) |  | 0.52 (0.23-1.14) | **0.32 (0.14-0.76)** |
| Fruits (≥2 times/d)**†** | ≤1 cigarette/d |  | Ref. | Ref. |  | Ref. | Ref. |
|  | 2-10 cigarettes/d |  | 1.08 (0.89-1.31) | 0.97 (0.55-1.70) |  | **0.70 (0.50-0.97)** | 1.04 (0.84-1.30) |
|  | >10 cigarettes/d |  | 1.00 (0.53-1.89) | 1.27 (0.36-4.50) |  | 0.91 (0.41-2.02) | 1.00 (0.46-2.16) |
| Vegetables (≥2 times/d)**†** | ≤1 cigarette/d |  | Ref. | Ref. |  | Ref. | Ref. |
|  | 2-10 cigarettes/d |  | 0.86 (0.71-1.03) | 0.87 (0.51-1.50) |  | 0.90 (0.67-1.22) | 0.85 (0.69-1.05) |
|  | >10 cigarettes/d |  | **0.46 (0.26-0.81)** | 0.42 (0.12-1.48) |  | 0.61 (0.29-1.29) | **0.34 (0.16-0.72)** |
| Milk (≥3 d/wk)**†** | ≤1 cigarette/d |  | Ref. | Ref. |  | Ref. | Ref. |
|  | 2-10 cigarettes/d |  | **0.68 (0.56-0.81)** | 1.03 (0.61-1.74) |  | **0.63 (0.47-0.85)** | **0.75 (0.61-0.92)** |
|  | >10 cigarettes/d |  | **0.42 (0.23-0.77)** | 1.89 (0.53-6.83) |  | 0.85 (0.40-1.79) | **0.36 (0.17-0.80)** |
| Soft drinks (≥1 times/d)**‡** | ≤1 cigarette/d |  | Ref. | Ref. |  | Ref. | Ref. |
|  | 2-10 cigarettes/d |  | **1.94 (1.58-2.38)** | 1.22 (0.56-2.66) |  | **2.43 (1.74-3.39)** | **2.46 (1.93-3.12)** |
|  | >10 cigarettes/d |  | **3.10 (1.74-5.52)** | **7.03 (1.99-24.86)** |  | **3.74 (1.75-7.98)** | **4.46 (2.15-9.24)** |
| Fast food (≥2 d/wk)**‡** | ≤1 cigarette/d |  | Ref. | Ref. |  | Ref. | Ref. |
|  | 2-10 cigarettes/d |  | 1.23 (0.97-1.55) | **1.91 (1.08-3.36)** |  | 0.97 (0.68-1.40) | 1.24 (0.96-1.61) |
|  | >10 cigarettes/d |  | **2.57 (1.40-4.72)** | 2.32 (0.66-8.18) |  | **2.48 (1.16-5.31)** | 1.96 (0.91-4.25) |
| **Physical Activity** |  |  |  |  |  |  |  |
| Moderate physical activity  (≥2d/wk)**‡** | ≤1 cigarette/d |  | Ref. | Ref. |  | Ref. | Ref. |
|  | 2-10 cigarettes/d |  | 0.98 (0.80-1.19) | **0.54 (0.32-0.94)** |  | 0.89 (0.64-1.22) | 1.04 (0.83-1.29) |
|  | >10 cigarettes/d |  | 1.44 (0.74-2.80) | 0.53 (0.15-1.90) |  | 0.88 (0.39-2.00) | 1.69 (0.73-3.93) |
| Muscle strengthening activity (≥2 d/wk)**‡** | ≤1 cigarette/d |  | Ref. | Ref. |  | Ref. | Ref. |
|  | 2-10 cigarettes/d |  | 1.00 (0.83-1.21) | 1.29 (0.68-2.44) |  | **1.89 (1.38-2.58)** | **1.41 (1.15-1.74)** |
|  | >10 cigarettes/d |  | 1.32 (0.74-2.35) | **8.11 (2.11-31.12)** |  | **3.73 (1.65-8.43)** | 1.58 (0.77-3.26) |
| Attend physical education classes (≥2 d/wk) | ≤1 cigarette/d |  | Ref. | Ref. |  | Ref. | Ref. |
|  | 2-10 cigarettes/d |  | **0.75 (0.59-0.94)** | 0.79 (0.42-1.49) |  | **0.56 (0.41-0.78)** | 0.91 (0.67-1.22) |
|  | >10 cigarettes/d |  | **0.50 (0.26-0.95)** | **0.25 (0.07-0.91)** |  | **0.29 (0.14-0.61)** | 1.00 (0.34-2.91) |
| **Sedentary Activity** |  |  |  |  |  |  |  |
| Watch TV (≥2 hours/d) | ≤1 cigarette/d |  | Ref. | Ref. |  | Ref. | Ref. |
|  | 2-10 cigarettes/d |  | 0.81 (0.66-1.00) | 1.03 (0.58-1.81) |  | 0.77 (0.54-1.09) | **0.73 (0.59-0.92)** |
|  | >10 cigarettes/d |  | 1.28 (0.71-2.31) | 1.98 (0.56-6.93) |  | 0.91 (0.39-2.12) | 1.74 (0.86-3.54) |
| Use computer (≥2 hours/d)**‡** | ≤1 cigarette/d |  | Ref. | Ref. |  | Ref. | Ref. |
|  | 2-10 cigarettes/d |  | **2.19 (1.79-2.68)** | **2.36 (1.27-4.41)** |  | **3.22 (2.25-4.60)** | **2.00 (1.60-2.50)** |
|  | >10 cigarettes/d |  | 1.33 (0.71-2.48) | 1.79 (0.36-8.80) |  | 1.61 (0.69-3.76) | 1.34 (0.60-3.01) |
| **Other** |  |  |  |  |  |  |  |
| Sleep duration (≥8 hours/d)**†** | ≤1 cigarette/d |  | Ref. | Ref. |  | Ref. | Ref. |
|  | 2-10 cigarettes/d |  | 0.95 (0.78-1.15) | 0.89 (0.52-1.53) |  | 1.14 (0.84-1.55) | 0.88 (0.71-1.09) |
|  | >10 cigarettes/d |  | 1.01 (0.55-1.86) | 1.17 (0.30-4.51) |  | 1.25 (0.57-2.71) | 0.91 (0.41-1.99) |
| Drink alcohol (yes)**†** | ≤1 cigarette/d |  | Ref. | Ref. |  | Ref. | Ref. |
|  | 2-10 cigarettes/d |  | **4.51 (3.73-5.44)** | **15.95 (7.44-34.20)** |  | **4.27 (3.12-5.85)** | **6.09 (4.88-7.60)** |
|  | >10 cigarettes/d |  | **4.86 (2.55-9.25)** | NA |  | **6.09 (2.41-15.38)** | **6.90 (2.93-16.25)** |
| Bold numbers represent significant results. NA: not available  **†** During the past 30 days. **‡** During the past 7 days. | | | | | | | |
